# Current treatments for non-small cell lung cancer

**DOI:** 10.3389/fonc.2022.945102

**Published:** 2022-08-11

**Authors:** Qianqian Guo, Liwei Liu, Zelong Chen, Yannan Fan, Yang Zhou, Ziqiao Yuan, Wenzhou Zhang

**Affiliations:** ^1^ Department of Pharmacy, Affiliated Cancer Hospital of Zhengzhou University & Henan Cancer Hospital, Zhengzhou University, Zhengzhou, China; ^2^ Department of Pharmacy, First Affiliated Hospital of Zhengzhou University, Zhengzhou, China; ^3^ Affiliated Cancer Hospital of Zhengzhou University & Henan Cancer Hospital, Artificial Intelligence and IoT Smart Medical Engineering Research Center of Henan Province, Zhengzhou, China; ^4^ Children’s Hospital Affiliated to Zhengzhou University, Henan Children’s Hospital, Zhengzhou Children’s Hospital, Zhengzhou, China; ^5^ Key Laboratory of Advanced Drug Preparation Technologies, Ministry of Education, School of Pharmaceutical Sciences, Zhengzhou University, State Key Laboratory of Esophageal Cancer Prevention and Treatment, Zhengzhou University, Zhengzhou, China

**Keywords:** NSCLC, diagnosis, chemotherapy, chemoradiotherapy, targeted therapy, antiangiogenic therapy, immunotherapy

## Abstract

Despite improved methods of diagnosis and the development of different treatments, mortality from lung cancer remains surprisingly high. Non-small cell lung cancer (NSCLC) accounts for the large majority of lung cancer cases. Therefore, it is important to review current methods of diagnosis and treatments of NSCLC in the clinic and preclinic. In this review, we describe, as a guide for clinicians, current diagnostic methods and therapies (such as chemotherapy, chemoradiotherapy, targeted therapy, antiangiogenic therapy, immunotherapy, and combination therapy) for NSCLC.

## 1 Introduction

Lung cancer, as a common malignant cancer, presents a serious threat to human life. Lung cancers can be divided into NSCLC and small cell lung cancer (SCLC), based on differences in histology and origin (1). NSCLC predominates, accounting for almost 85%, of lung cancer cases. NSCLC is further subdivided into two main subtypes: lung adenocarcinoma (LUAD) and lung squamous cell carcinoma (LUSC). The two types have different gene expression profiles, especially of *NECTIN1*, a cadherin biomarker (2). In addition, LUSC proliferates faster than LUAD (3).

The causes of lung cancer are diverse, but smoking is considered to be the primary reason. In some lung cancer patients with no smoking history, the disease can be attributed to exposure to radon (^222^Rn), usually from building materials (4). The incidence of lung cancer is also related to genetics and demographic characteristics (5). The link with demographic characteristics may be attributable to differences in health care systems in different countries. For example, differences in the physical examination of patients may affect the stage at which lung cancer is diagnosed (the development of NSCLC can be divided into four stages: I, II, III, and IV) (6). The main reason for the high mortality rate among lung cancer patients is that only 15% of patients are diagnosed at an early stage (7), and in most patients (70%) the disease is not diagnosed until it is at an advanced stage, perhaps because symptoms are relatively slight in the early stages, and patients may ignore them.

It appears that NSCLC does not metastasize in the early stages and, therefore, surgery could extend the life of patients provided the disease is diagnosed at this stage (8). However, surgery will not benefit those patients, the majority, in whom the disease is diagnosed at an advanced stage. Therefore, the low rate of diagnosis of NSCLC in the early stages remains a problem.

The use of positron emission tomography (PET) could increase the proportion of patients in whom lung cancer is diagnosed in the early stages and thereby reduce lung cancer mortality. The problem is how to increase the number of patients who undergo PET. Common symptoms of lung cancer, such as coughing, chest pain, and wheezing, are often ignored by patients, and hemoptysis, although more likely to be worrying to patients, is experienced by only 20% of lung cancer patients (9). As a result, many patients miss out on the opportunity for early diagnosis and effective treatment.

**Figure 1:**
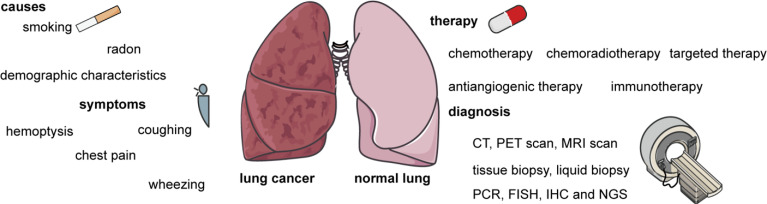
The main causes and symptoms of lung cancer, as well as methods of diagnosis and therapies. The causes of lung cancer include smoking, radon, genetics, and demographic characteristics. The symptoms of lung cancer including hemoptysis, coughing, chest pain, and wheezing. Therapies include chemotherapy, chemoradiotherapy, targeted therapy, antiangiogenic therapy, immunotherapy, and combination therapy. Diagnostic methods include computed tomography (CT), positron emission tomography (PET), magnetic resonance imaging (MRI), tissue biopsy, liquid biopsy, polymerase chain reaction (PCR), fluorescence *in situ* hybridization (FISH), immunohistochemistry (IHC), and next-generation sequencing (NGS).

Treatments for lung cancer include chemotherapy, chemoradiotherapy, targeted therapy, antiangiogenic therapy, immunotherapy, and combination therapy. Treatment of stage II–IV disease also involves adjuvant therapy and neoadjuvant therapy, in addition to the therapies mentioned above. In some cases, these therapies can be used to confirm the success or otherwise of surgery or combined with surgery to give better results. Besides, surgery is the main treatment for stage I disease. In this review, we describe the biological features of lung cancer, diagnostic methods, and drugs or other compounds currently used in chemotherapy, chemoradiotherapy, targeted therapy, antiangiogenic therapy, immunotherapy, and combination therapy (**Figure 1**). We hope that this review will act as guidance for the clinical treatment of lung cancer.

## 2 The biological features of lung cancer

Lung cancer is a heterogeneous cancer, which means that the tumor contains different subpopulations of cells. Heterogeneity is correlated with chemoresistance and the probability of metastasis (10). Diagnostic methods, therapeutic methods, and the identification of novel biomarkers would also benefit from the further study of lung cancer biology. It is therefore important to summarize the biological features of lung cancer.

### 2.1 Oncogene mutations in NSCLC patients

Oncogene mutations are found in most NSCLC patients and, therefore, targeted drugs are associated with fewer side effects, higher response rates (RRs), and longer progression-free survival (PFS) than cytotoxic drugs. A mutation in the gene coding for *epidermal growth factor receptor (EGFR)* is common in NSCLC patients (found in 10%–30% of patients), and downstream signaling pathways such as MAPK/ERK, PI3K/AKT and Bax/Bcl-2 are also potential targets (11). Almost 90% of *EGFR* mutations in NSCLC patients are exon 19 deletions or *L858R* substitutions in exon 21. In addition, mutation of the *T790M* gene occurs in 50%–60% of NSCLC patients with the *EGFR* mutation, and this mutation is associated with acquired resistance (12). Acquired resistance to the EGFR tyrosine kinase inhibitor (TKI) in NSCLC patients is correlated with overexpression of osteopontin (OPN), upregulation of integrin αVβ3, and activation of downstream signaling pathways such as FAK/AKT and ERK (13). Activation of the PI3K/AKT/mTOR signaling pathway is also associated with acquired resistance to *EGFR* TKIs in NSCLC patients (14). The PI3K/AKT/mTOR signaling pathway is linked to the proliferation and invasion of cancer cells, affecting the likelihood of success of chemotherapy.

*Rearranged during transfection (RET)* rearrangements are found in 1%–2% of NSCLC patients, and the downstream signaling pathways of *RET*, such as PI3K/AKT, JAK-STAT, and RAS/MAPK, are associated with cell proliferation, invasion, and migration (15, 16). *MET* mutations could result in the abnormal expression of MET axis, and the MET/HGF (hepatocyte growth factor) signal pathway play an important role in the MET axis, and this signal pathway leads to tumor cell migration, invasion, and metastasis (17) and are associated with resistance to treatment with *EGFR* and vascular endothelial growth factor receptor (VEGFR) inhibitor. Mutations in exon 14 are the most common *MET* mutations found in NSCLC patients (18). The majority of *MET* exon 14 mutations are point mutations, but indels, insertions, and deletions are also found (19).

Rearrangement of the anaplastic lymphoma kinase gene (*ALK*) has been identified in 5%–6% of younger NSCLC patients (20). Overexpression of ALK in A549 cells can induce epithelial–mesenchymal transition (EMT), and increase migration and invasion, phenomena that are correlated with the upregulation of signal transducer and activator of transcriptions 3 (STAT3) (21). Many NSCLC patients with an *ALK* mutation develop drug resistance after taking drugs for a few years. In the case of ALK inhibitors, the most common mutation associated with acquired resistance is F1174L (22). In addition, some studies have confirmed that drug resistance in NSCLC is associated with signal transducer and activator of transcriptions (STATs), especially the STAT3/ZEB1 signaling pathway (23). These findings are a reminder that combination therapies targeting both ALK and STAT3 could perhaps overcome the resistance associated with the use of ALK inhibitors.

Mutations in the gene encoding human epidermal growth factor receptor 2 (*HER2*) is the mutation of exon 20, and these mutations are found in 2%–4% of NSCLC patients, especially women, besides, the patients with *HER2* mutations easily appear brain metastases (24). Activation of *HER2* induces the phosphorylation of tyrosine residues, leading to the activation of downstream signaling pathways such as MEK/ERK and PI3K/AKT, which in turn increases the migration and proliferation of lung cancer cells (25). Around 4% of NSCLC patients have a mutation in the *B-Raf proto-oncogene* (*BRAF*), but the *V600E* mutation is present in only half of such patients, who as a result are resistant to *BRAF* inhibitors (the *V600E* mutation is associated with a better response to *BRAF*-targeted therapy) (26). *BRA*F*^V600E^* mutation is usually accompanied by MAPK signaling pathway activation, and, therefore, combination therapy with two different drugs, one targeting *BRAF* and the other targeting *MEK (* 27), may give better results.

*c-Ros oncogene 1* (*ROS1*) rearrangement is found in around 1%–2% of NSCLC patients (28). There are several different ROS1 rearrangements, including *CD74-ROS1, SLC34A2-ROS1, YWHAE-ROS1, TFG-ROS1*, and *CEP85L-ROS1*, but *CD74-ROS1* (44%) is the most common *ROS1* rearrangement found in NSCLC patients (29). ROS1 is a kind of tyrosine kinase; its ligand is neural epidermal growth factor-like 2 neural EGFL-like 2 (30). Just as the other oncogene we mentioned above, such as HER2, BRAF, when ROS1 is activated by its ligands, downstream signaling pathways such as the PI3K/AKT/mTOR, JAK/STAT, and MAPK/ERK signaling pathways are also activated, leading to the proliferation of lung cancer cells and tumor invasion (31).

Among NSCLC patients tested, 13% were found to have the *Kirsten rat sarcoma viral oncogene* (*KRAS*) *p.G12C* mutation (32). *KRAS* mutations, like mutations of other oncogenes, are associated with drug resistance and poorer outcomes in NSCLC (33).

*KRAS* mutations are also known to be present in 90% of smokers. *KRAS* is related to inflammation, and *KRAS* mutation is found in the most smokers, therefore, it may be some sort of inflammatory reaction in lung cells by smoking (34). The drug resistance induced by *KRAS* mutations is usually intrinsic. However, *KRAS* mutations are heterogeneous, i.e., there is more than one type, and different *KRAS* mutations lead to activation of different downstream signaling pathways. *KRAS* mutations do not result in changes in the phosphorylation of the AKT signaling pathway (35).

**Figure 2:**
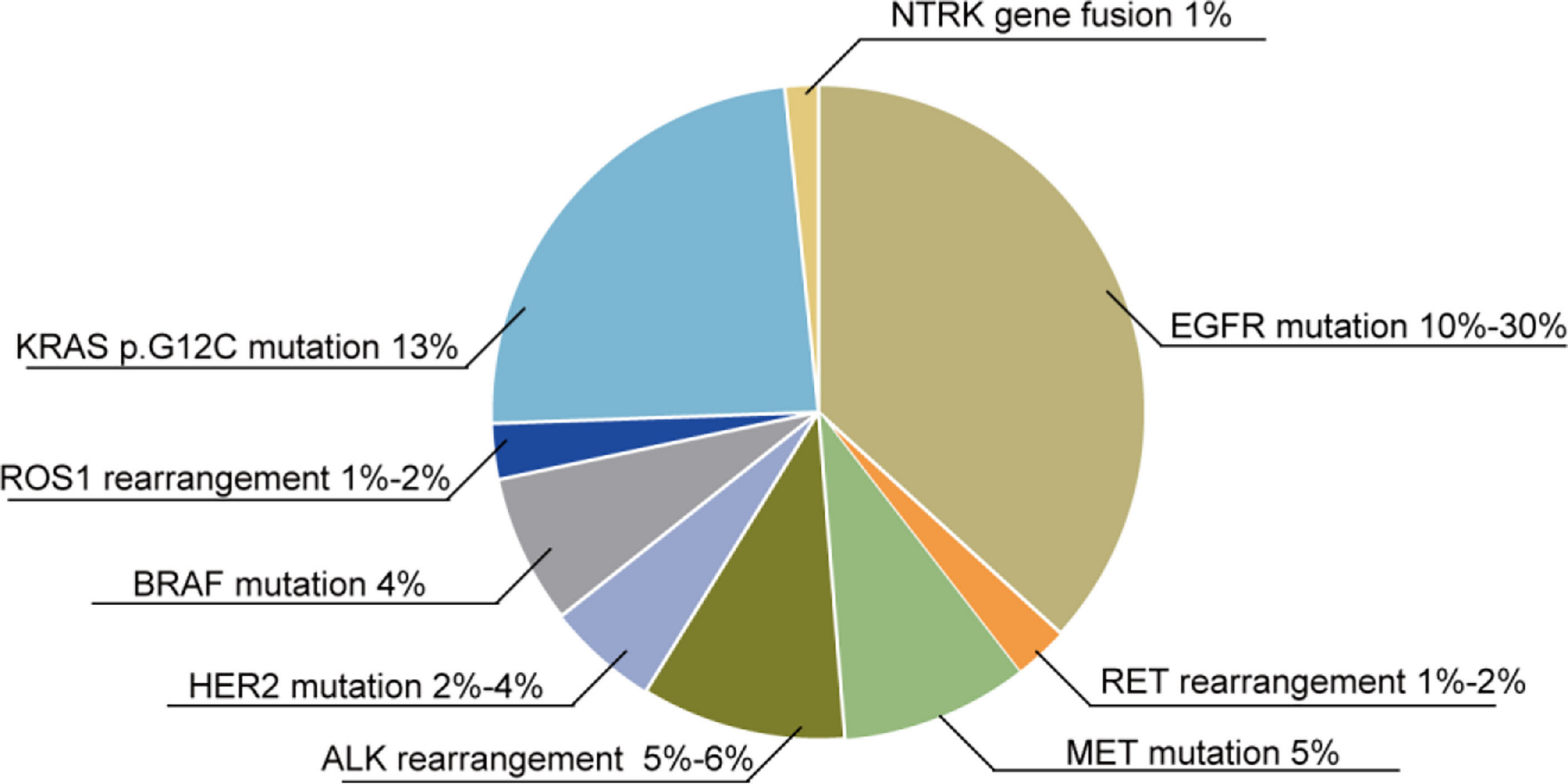
Oncogene mutations in NSCLC patients. Various oncogene mutations are found in NSCLC patients: 10%–30% of NSCLC patients exhibit *EGFR* mutations, 1%–2% have *RET* rearrangements, 5% have a *MET* mutation, 5%–6% have an *ALK* rearrangement, 2%–4% have a *HER2* mutation, 4% have a *BRAF* mutation, 1%–2% have *ROS1* rearrangements, 13% have the *KRAS p.G12C* mutation, and 1% have *NTRK* gene fusions.

Fusion of the *neurotrophic tropomyosin receptor kinase (NTRK)* gene is a relatively rare oncogene mutation, which occurs in less than 1% of NSCLC patients. The detection of *NTRK* fusions relies on RNA-based next-generation sequencing (NGS) (36). The downstream signaling pathways include the MEK/ERK and PI3K/AKT signaling pathways. As mentioned above, these signaling pathways are related to cancer cell proliferation and migration, and the PI3K/AKT signaling pathway is also involved in apoptosis, which is induced by chemotherapy (36). In early-stage NSCLC with *NTRK* gene fusions (**Figure 2**), patients have a high RR to TKIs (37).

Moreover, the immune checkpoint development also benefits NSCLC patients. If the mutation in patients does not concern the mutations above, then the *programmed death ligand 1 (PD-L1)* mutation maybe a better choice, but there are still some limits, for example, the mutation of PD-L1 at least appears 50% mutation in the lung cancer patients (38). The combination of programmed death 1 (PD-1) and PD-L1 would decrease immune response; therefore, the tumor cells will escape the surveillance of immune cells such as T cells. There are also some studies reported that the *EGFR* mutation in NSCLC could increase the expression of PD-L1 protein, and TKIs could reduce the amount of PD-L1 protein, the signaling pathways referring to this phenomenon are PI3K-AKT, STAT3, NF-κB, and MEK-ERK signaling pathways (39). Furthermore, *ALK* and *KRAS* mutations could improve the expression of *PD-L1*; therefore, if the patients are harboring *PD-L1* and *EGFR* or *ALK* or *KRAS* at the same time, patients will have a higher RR when the interaction between PD-1 and PD-L1 is blocked (40). However, there is still no study that can verify the results for NSCLC patients harboring several mutations at the same time. The high PD-L1 expression is also associated with smoking, and PD-L1 usually appears in the early stage of NSCLC, and could become a biomarker in the diagnosis of lung cancer (41).

Apart from the targets mentioned above, there are also some signaling pathways abnormally expressed in NSCLC that could become new biomarkers in diagnosis and therapy, but these signaling pathways still stand in the preclinical stage.

### 2.2 long non-coding RNAs, microRNAs, and abnormal proteins in NSCLC

The long non-coding RNAs (lncRNAs) and microRNAs (miRNAs) are non-coding RNAs existing in the cells, and these non-coding RNAs are correlated with tumor progression and tumor features, for example, its proliferation, migration, invasion, resistance, and recurrence. In NSCLC, these non-coding RNAs also show a more important role, and some results in preclinical studies could give rise to new biomarkers or targets in the diagnosis and treatment of NSCLC.

#### 2.2.1 lncRNAs and NSCLC

lncRNA H19 and miRNA-21 overexpress in the NSCLC tumor, and these could become biomarkers in the diagnosis and treatment of NSCLC (42). Circular RNAs (circRNAs) hsa_circ_0058357 overexpress in NSCLC, and the abnormal expression of hsa_circ_0058357 is associated with migration, proliferation, and apoptosis through increasing AVL9 accompanied by the inhibition of miR-24-3p (43). LncRNA SNHG14 is a cancer-promoting lncRNA, and it is upregulated in the lung cancer tissue; lncRNA SNHG14 could promote the migration, proliferation, and invasion of NSCLC cells; and lncRNA SNHG14 could inhibit the miR-206 expression; therefore, the downstream targets of miR-206 such as G6PD are upregulated (44). lncRNA ABHD11-AS1 is overexpressed in NSCLC, and it could upgrade the Warburg effect and proliferation of NSCLC. There is m6 A methyltransferase-like 3 (METTL3) in the upstream of lncRNA ABHD11-AS1, which could promote the expression of ABHD11-AS1, and the prognosis for NSCLC patients will get worse (45). lncRNA DUXAP8, an oncogenic lncRNA, could induce the proliferation, EMT, and aerobic glycolysis in lung cancer cells. Its effects will be studied further. Moreover, the overexpression of lncRNA DUXAP8 in NSCLC patients is correlated with the poor prognosis. The mechanisms here are diverse including transcriptional, post-transcriptional, and epigenetic regulation (46). The overexpression of lncRNA CCDC144NL-AS1 in NSCLC patients could promote the proliferation, migration, and invasion of NSCLC cells (H1299, A549, NCI-H650, and HCC827 cells). Mechanically, lncRNA CCDC144NL-AS1 could directly bind to miR-490-3p (47). There are also some other examples showing that lncRNA could be a biomarker in NSCLC, one is LncRNA HOTAIR that could promote the proliferation, invasion, and migration in NSCLC cells by regulating the CCL22 signaling pathway (48). lncRNA UFC1 could promote the progression of NSCLC by downregulating the expression of PTEN through zeste homolog 2 (EZH2) (49). LncRNA WTAPP1 could promote the invasion and migration of NSCLC cells by suppressing the expression of lncRNA HAND2-AS1 (50).

In addition, there are also some lncRNAs that play an inhibitor role in the progression of NSCLC. LncRNA NBR2 is downregulated in NSCLC patients, and the overexpression of lncRNA NBR2 could inhibit the migration of lung cancer cells (SPC-A1 cells) and the Notch signaling pathways are also suppressed, and the EMT-related genes are also reduced (51). lncRNA LINC00261 is downregulated in the lung cancer tissues, and the overexpression of lncRNA LINC00261 in A549 and SPC-A1 cells would inhibit metastasis *in vitro* and *in vivo* through regulating the miR-1269a/FOXO1 signaling pathway (52). There is a novel lncRNA BRCAT54 that is overexpressed in the lung cancer tissue, but this lncRNA benefits the patients, and its knockdown could promote the migration, proliferation, and apoptosis inhibition of lung cancer cells, which concern the regulation of JAK-STAT and calcium-related signaling pathways (53).

#### 2.2.2 microRNAs and NSCLC

The microRNA functions in NSCLC are different. Some microRNAs show promotion in the progression of lung cancer, and others show inhibition in the progression of lung cancer.

Radiotherapy is useful for most NSCLC patients in the early stage, but radiotherapy is usually accompanied by acquired resistance. Acquired resistance has been proven to be correlated with the overexpression of miR-410 in NSCLC. Mechanically, miR-410 could induce EMT and target the PTEN/PI3K/mTOR signaling pathway (54). miR-10b aberrantly expresses in multiple malignant cancers, such as breast cancer, esophageal cancer, pancreatic cancer, and lung cancer, and it is related to proliferation and invasion (55). miRNA-21 is overexpressed in NSCLC and is related to the poor survival and prognosis of patients, especially with miRNA-21 being correlated with the radiation resistance of NSCLC. Therefore, the inhibition of miRNA-21 in NSCLC cells (A549 cells) could suppress proliferation and improve sensitivity to radiation through increasing apoptosis (56). miR-142-3p, on the one hand, could improve the sensitivity of NSCLC by downregulating the high-mobility group box-1 (HMGB1) protein and inhibiting autophagy. On the other hand, it could also play as an oncogene, and its overexpression is correlated with the poor outcome of NSCLC patients in clinical treatment, and promotes the migration and proliferation of NSCLC cells by downregulating TGFβR1 (57).

In the NSCLC tissues, miR-936 is at a low expression, and the overexpression of miR-936 could block the cell cycle, and inhibit the proliferation and invasion of NSCLC cells. At the same time, the downstream target E2F transcription factor 2 (E2F2) that could promote the invasion of NSCLC is downregulated (58). The overexpression of miR-221–3p could decrease the resistance of paclitaxel by inducing apoptosis accompanied by the inhibition of MDM2/p53 signaling pathway (59). miR-340 is at a lower expression of NSCLC tissues, and its overexpression could inhibit the migration and invasion of NSCLC cells through targeting RAB27B. In addition, the overexpression of miR-340 could suppress proliferation and induce apoptosis through regulating p27 (60). The level of miRNA-597 in the NSCLC tissues is lower than the normal tissue, and the downregulated miRNA-597 is related to the stage and poor prognosis of NSCLC patients. The overexpression of miRNA-597 could inhibit progression by regulating CDK2 (61). miR-4732-5p expression is inhibited in NSCLC; its downregulation is related to metastasis, late stage, and poor outcome of NSCLC patients. Its overexpression could suppress the proliferation, migration, and invasion of NSCLC cells (A549, HCC827, H23, and H1975 cells) by regulating TSPAN13 (also known as NET-6 and TM4SF13) that has been proven to inhibit proliferation and invasion in breast cancer (62, 63).

#### 2.2.3 Abnormal proteins and NSCLC

There are also some proteins that overexpress in the NSCLC patients, which could become new targets in clinical trials. Fibulin2 (FBLN2) is decreased in the lung cancer cell lines, and the overexpression of FBLN2 would inhibit the activation of MAPK/ERK and AKT/mTOR signaling pathways, accompanied by the decreased migration and invasion of cells (64). This means that FBLN2 could be a potential biomarker for detecting NSCLC in the clinic. The abnormal expression of nuclear factor kappa B (*NF-κb*) is correlated with chemoresistance and radio-resistance in lung cancer therapy, and the inhibition of NF-κβ signaling pathway will decrease the resistance given by chemotherapy and radiotherapy (65). *NF-κb* is related to multi-signaling pathways such as apoptosis, angiogenesis, and inflammation; therefore, *NF-κb* is a relatively difficult oncogenic mutation compared with other oncogene mutations such as *EGFR* and *KRAS* (66). Nuclear factor erythroid 2-related factor 2 (Nrf2) is increased and Keap1 in cytoplasmic is decreased, and these changes in Nrf2 and Keap1 are correlated with the poor outcome of NSCLC patients, and increased Nrf2 may contribute to chemoresistance when using platinum-related chemotherapy (67). Almost 25% of patients with NSCLC appear to have brain metastases, and there are several aberrant proteins arising in this process. NFATc1 and NFATc3 are listed in these biomarkers, and the expression of these two proteins is decreased in patients with brain metastases, at the same time, the downstream targets such as IL-11 (correlated with JAK-STAT3 signaling pathways), CDH5 (correlated with metastasis), and CCL2 (correlated with proliferation and apoptosis) are also regulated by NFATc1 and NFATc3 (68). Tripartite motif (TRIM) protein is a type of protein correlated with multiple malignant cancers including lung cancer, and takes part in various signaling pathways regulation including p53, NF-κB, and PI3K/AKT. In NSCLC, TRIM could play as an oncogene or suppressor. As disintegrins and metalloproteinases with thrombospondin motifs (ADAMTS8) are downregulated in NSCLC cells (H460 and A549 cells), the overexpression of ADAMTS8 could inhibit proliferation and induce apoptosis of lung cancer cells. Mechanically, the vascular endothelial growth factor A (VEGFA) and CD31 are suppressed (69). Neurexophilin 4 (NXPH4) is overexpressed in NSCLC tissues, and its knockdown could suppress the proliferation and migration of NSCLC cells (A549, H226, H2106, and HCC827 cell line), and trigger cell cycle arrest in phase S1. EZH2 was in the upstream of NXPH4, and could activate the expression of NXPH4; then, the activated NXPH4 could downregulate the expression of CDKN2A, and the downregulated CDKN2A could regulate the cyclinD-CDK4/6-pRB-E2F signaling pathway resulting in the cell cycle activation and the promotion of proliferation and migration of lung cancer cells (70).

### 2.3 CSCs and lung cancer

Cancer stem cells (CSCs) are considered to be the root of cancer, and evidence confirm that CSCs are related to chemoresistance and recurrence and the survival of lung cancer patients. Therefore, there are many compounds targeting CSCs in preclinical or clinical trials. There are also other strategies that inhibit the stemness of cancer cells. More specifically, targeting signaling pathways such as Wnt, hippo, and notch could inhibit the stemness of cancer cells or the biomarkers correlated with CSCs (71). CSCs also exist in NSCLC, and lung cancers also have the feature of stemness; therefore, these facts confirm that targeting CSCs in NSCLC is crucial (72, 73).

Lung cancer stem cells (LCSCs) with high chemo-resistance were obtained from the NSCLC patients; the subpopulation of LCSCs show self-renewal, resistance, invasion, and tumorigenic potential in the *in vitro* experiments, and the CDKN1A, ITGA6, and SNAI1that were selected by different expression levels between LCSCs and the adherent-cultured cells could become biomarkers for indicating the different stages of lung cancer in patients (74). The LCSC biomarkers in humans include CD133^+^, CD90^+^, CD44^+^, CD87^+^, ABCG2, SP, and ALDH (75). Forkhead box C1 (*FOXC1*) is correlated with the CSC features, and is elevated in NSCLC. The knockdown of FOXC1 could decrease the subpopulation of CD133^+^ cells, and the associated genes, such as *NANOG, ABCG2, SOX2*, and *Oct4*, are also downregulated, and the chemo-sensitivity for cisplatin, docetaxel, and gefitinib is also increased (76). m6A demethylase *ALKBH5* is upregulated in LCSCs, and its knockdown could contribute to the E-cadherin upregulation and stem markers such as NANOG and Oct4 are downregulated. Mechanically, there is a positive relationship between *ALKBH5* and *p53*, and the knockdown of *p53* would make *ALKBH5* downregulate, and the tumor formation ability and invasion are also suppressed (77). Nerve injury-induced protein 1 (Ninj1) is upregulated in NSCLC cells and tissues; the subpopulation of Ninj1^high^ LCSCs exhibits the CSC-related features such as the increase of ALDH^+^ subpopulation, sphere-forming ability, and stemness markers; and the downstream signaling pathway Wnt/β-Catenin is also activated by Frizzled2-LRP6 assembly (78). Histamine N-methyltransferase (HNMT) is overexpressed in NSCLC tissues as found in clinical trials, and is related to a poor prognosis for patients. Moreover, HNMT has a positive relationship with HER2 that could improve the features of CSCs. The knockdown of HNMT could decrease the tumorsphere formation ability, and reduce the expression of CSC markers such as NANOG, CD133, OCT4, and KLF4 through the Nrf2/HO-1/HER2 signaling pathway increasing the accumulation of reactive oxygen species (ROS) (79). The stemness markers ALDH and CD133 are well-verified in LCSCs; p53 is a cancer suppressor, the mutation which is found in 47% of NSCLC cases, and the knockdown of the three genes could reduce the CSC characteristics and prolong the survival of NSCLC patients (80). This study is a reminder that the stemness markers may have some therapeutic effect in NSCLC patients. Heat shock protein 90 (hsp90) inhibitors show better results in clinical use, but in therapy, there is resistance that maybe correlated with CSCs in lung cancer. However, there is a new Hsp90 inhibitor named NCT-80 that could reverse CSCs resulting to resistance by regulating STAT3/Wnt/β-catenin signaling pathways (81). RNF168, a E3 ubiquitin ligase, is downregulated in lung adenocarcinoma, but upregulated in squamous cell carcinoma; the overexpression of RNF168 could inhibit the CSC features (such as sphere-formation ability, stemness markers ALDH) of NSCLC cells. Mechanically, the RNF168 could ubiquitylate RhoC and cause its degradation (82). Non-muscle myosin heavy chain 9 (MYH9) is upregulated in lung cancer, and correlated with the worst prognosis in NSCLC patients, and the overexpression of MYH9 in lung cancer cells could improve the expression of stemness markers (such as SOX2, OCT4, Nanog, CD133, and CD44) and sphere-formation ability by regulating the mTOR signaling pathway (83). The Orai3 channel is a calcium channel related to the chemoresistance of lung cancer, and the overexpression of Orai3 could improve metastasis in NSCLC. LCSCs, derived from NSCLC cells with cisplatin resistance, has a higher expression of Orai3, and the silence of Orai3 could worsen metastasis, accompanied by a sensitivity to cisplatin. Moreover, stemness markers such as Sox2 reduced through regulating the PI3K/AKT signaling pathway (84).

Overall, there are still many stemness markers of NSCLC studied in the preclinical and clinical trials, and the development of small molecular markers could become the new targets or diagnostic markers for the different stages of lung cancer.

### 3 The diagnosis of lung cancer

Except for the symptom of coughing appearing in the early stage of lung cancer, most lung cancer patients are asymptomatic in the early stage; therefore, early diagnosis and treatment could be missed. The development of technology in diagnosis could save majority of patients and could prolong their lives. Diagnostic methods mainly include image test, biopsy test, and biomarker test.

#### 3.1 Image test

Image tests, such as computed tomography (CT), PET scan, and magnetic resonance imaging (MRI) scan, play an important role in the diagnosis of lung cancer. CT is the most common diagnostic means in lung cancer, which could determine tumor size (≥ 6 mm) and the number of nodules in lung cancer patients. It also could test the metastases, especially the mediastinal lymph nodes in the lung cancer patients (85–87). CT could also detect if the nodules are benign or malignant, but for further determination, biopsy is still needed (88). PET has more sensitivity and specificity than CT because the PET scan uses fluorine-18 fluorodeoxyglucose (F-18 FDG) as the biomarkers. It could locate in the malignant lesions with aberrant glucose metabolism (89). PET could also test if the lesions are benign or malignant, and it also differentiates the different types and staging (especially the distant metastases) of lung cancer by the uptake degree of FDG (90, 91). MRI scan has been used in NSCLC patients with brain and bone metastases because the dye used in MRI scan is not suitable for tissues that can move. With the development of high-performance gradient systems, phased-array receiver coil, and optimized imaging sequences, MRI could also detect nodules in lung tissues; the lowest size of nodules that can be detected is 3 mm (92).

#### 3.1.2 Biopsy test

Furthermore, the identification of lung cancer also needs biopsy (93), which could be tissue or liquid biopsy. Tissue biopsy is a type of invasive mean, and liquid biopsy is a non- invasive mean. Tissue biopsy is the gold standard to test lung cancer in the clinic. The determination of different histological types of lung cancer relies on tissue biopsy (94). Tissue biopsy could also test the mutations in lung cancer, but lung biopsy usually has complications (95). With the limitations of liquid biopsy, its application is restricted. In liquid biopsy, the sample used is the peripheral blood of the NSCLC patients, and the common testing indicators are circulating tumor DNA (ctDNA), circulating tumor cells (CTCS), and exosomes (96). In addition, it could also detect miRNA, circRNAs, circulating tumor vascular endothelial cells (CTECs), and tumor-educated blood platelets (TEPs) (97). Compared with tissue biopsy, liquid biopsy is more sensitive, effective, practical, and acceptable, and it could provide different mutations in the tumor (98).

#### 3.1.3 Biomarker test

Regarding the development of targeted therapy in NSCLC, if patients are diagnosed with NSCLC, then they are advised to take molecular testing to verify possible mutations. The methods are diverse. For example, polymerase chain reaction (PCR) could identify signal gene mutation, mostly used in determining the mutation of *EGFR* in the clinic (99). Fluorescence *in situ* hybridization (FISH) was approved by the food and drug administration (FDA) to test *ALK* rearrangements by fixing the tissue in formalin and embedding in paraffin (20). FISH could also diagnose the aberrant expression of *ROS1, RET, HER2, and MET (* 100). Immunohistochemistry (IHC) analysis is suitable for testing the mutations of *PD-L1* (approved by FDA), *ROS1, EGFR, BRAF-V600E*, and *RET (* 101). Moreover, IHC could be used in testing the mutation of *ALK* (approved by FDA) (102). NGS is suitable for almost all of mutations appearing in the NSCLC, such as *EGFR, RET, MET, ALK, HER2, BRAF, ROS1, KRAS*, and *NTRK*, also including some new biomarkers such as *PIK3CA (* 103). The NGS efficiency is high, the needed sample is small, and the cost is relatively low; therefore, there are more applications of NGS in the clinic.

The development of diagnostic methods in lung cancer (**Table 1**) could help most patients diagnosed in the early stage; therefore, the treatments for lung cancer could work.

**Table 1:** Diagnostic methods in lung cancer.

	Diagnostic method	Details
Image test	CT	Determines the size (≥ 6 mm) and number of nodules
	PET scan	With more sensitivity and specificity than CT, using F-18 FDG
	MRI	Used in NSCLC patients with brain and bone metastases, the lowest size of nodules could be 3 mm
Biopsy test	Tissue biopsy	Invasive mean, could test mutations
	Liquid biopsy	Non-invasive mean, testing indicators: ctDNA, CTCS, miRNA, circRNAs, CTECs, TEPs, and exosomes
Biomarker test	PCR	Determines the mutation of *EGFR*
	FISH	Tests the mutation of *ALK, ROS1, RET, HER2*, and *MET*
	IHC	Tests the mutation of *PD-L1*, *ROS1, EGFR, BRAF-V600E, ALK*, and *RET*
	NGS	Tests the mutation of *EGFR, RET, MET, ALK, HER2, BRAF, ROS1, KRAS*, PIK3CA, and *NTRK*

## 4 Treatments for lung cancer

### 4.1 Chemotherapy and chemoradiotherapy

#### 4.1.1 Chemotherapy

Before targeted therapy, chemotherapy dominated the clinical treatment for lung cancer. After the gene types of NSCLC have been identified in the clinic, chemotherapy was gradually replaced by targeted therapy, but chemotherapy also concerns cisplatin combination therapy. Currently, chemotherapy in NSCLC mostly involves cisplatin and carboplatin plus gemcitabine, taxanes, and pemetrexed plus some targeted therapy drugs such as VEGFR inhibitor (bevacizumab) or EGFR inhibitor (erlotinib) (104). The mechanism of chemotherapy is diverse. Cisplatin, carboplatin, and gemcitabine could disturb the DNA repair system, create DNA damage, and induce apoptosis in the cancer cell (105, 106). Taxanes could interfere with microtubule dynamics, trigger cell cycle arrest, and induce apoptosis (107, 108). Pemetrexed, an antifolate drug, could cause cell cycle arrest in the S phase (109).

The limitations of chemotherapy in lung cancer treatment mainly involve intrinsic resistance even though the compounds could have some effects at the first early treatment, but the tumor can acquire resistance rapidly (110). This disturbs the process of chemotherapy in the lung cancer treatment. There are various mechanisms of resistance in lung cancer. CSCs are correlated with the resistance of chemotherapy and radiation therapy as some compounds directly targeting CSCs could reduce the resistance in lung cancer therapy and improve the outcome of chemotherapy and radiosensitivity. The compounds target CSCs, mostly targeting the representative signaling pathways in the CSCs, such as Notch, MYC. RO4929097 (an inhibitor of Notch signaling pathway, γ-secretase inhibitor) combined with erlotinib could improve the efficiency of erlotinib in advanced NSCLC with chemoresistance and the PFS was up to 5 years (*NCT01193881 (first posted: 2 September 2010), NCT01193868 (first posted: 2 September 2010)*). In preclinical research, sulforaphane could inhibit the properties of LCSCs, such as sphere-forming ability, biomarkers of LCSCs, which could combine with cisplatin and doxorubicin to reduce the chemoresistance of NSCLC (111). Additionally, there are also some signaling pathways related to the resistance of lung cancer, which could provide a combined strategy for chemotherapy to overcome the resistance further. For example, Acetyl-11-keto-β-boswellic acid (AKBA) could improve the sensitivity of cisplatin in NSCLC through targeting P21, which maybe correlated with the increase of apoptosis and the inhibition of autophagy (112). This study reminds us that AKBA could become a new combination therapy in the clinic, even though it is still in preclinical research. The regulation of cell death such as autophagy, apoptosis, and ferroptosis could provide a new perspective to reducing resistance in chemotherapy (71).

Moreover, chemotherapy and radiotherapy have a function in neoadjuvant or adjuvant therapy in stage III NSCLC patients. Chemotherapy could help ensure that surgery goes well and could also serve as supplement after surgery (113). For example, patients with nodal metastases after surgery could benefit from adjuvant cisplatin-based therapy, and induction therapy could serve as a precondition for surgery (114, 115).

#### 4.1.2 Chemoradiotherapy

Radiotherapy is usually used in the local control of different stages of lung cancer, especially stage III unresectable NSCLC, which accounts for 30% in NSCLC patients (104). Moreover because of the development of four-dimensional computed tomography (4DCT), stereotactic body radiotherapy (SBRT), and intensity-modulated radiotherapy (IMRT), the side effects of radiotherapy are reduced (116). However, even though radiotherapy is the standard therapy for stage III NSCLC patients, the survival rate of patients has not improved. After the application of sequential radiotherapy to patients, the overall survival (OS) improved, but elderly patients still have not benefited from it. Therefore, combination therapy with radiotherapy may be of benefit to diverse patients with different states of health (117).

The mechanism of radiotherapy is mainly the damage of DNA, and damaged DNA could induce immune responses in the lung cancer; therefore, the combination therapy of radiotherapy and immunotherapy could produce a better result in the treatment of lung cancer (118). This combination has been verified by clinical trials. For example, in a phase III trial (*NCT02125461 (first posted: 29 April 2014)*), the conventional chemoradiotherapy (platinum-based chemotherapy and radiotherapy) plus durvalumab (an immune checkpoint inhibitor of PD-L1) could significantly prolong OS (up to 4 years) in stage III NSCLC patients compared with chemoradiotherapy alone, and the PFS of patients was also up to 3 years (119).

Chemoradiotherapy (CRT) mostly adjusts to the limited-stage SCLC. In addition, CRT also offers benefit for the lung cancer without metastasis. The chemotherapy in chemoradiotherapy generally includes cisplatin–etoposide (120) and carboplatin plus etoposide (121).

### 4.2 Targeted therapy

The lung cancer is driven by mutation of multiple oncogenes, the targetable alterations in the clinic provide probability for targeted therapy (122). In order to conduct targeted therapy in lung cancer patients, the molecular mutations in the tumor must be confirmed by diagnostic assays (123). The development of NGS provides a method to test the mutations appearing in lung cancer patients, which could help them get precision and personalized treatment in the clinic (124).

#### 4.2.1 Drugs approved by FDA

The targets that have drugs approved by FDA include *EGFR* (gefitinib (brand name: Iressa, company: ASTRAZENECA,London, the UK), erlotinib (brand name: Tarceva, company: OSI PHARMS, Ardsley, the USA), afatinib (brand name: Gilotrif, company: BOEHRINGER INGELHEIM, southwest Washington, the USA), dacomitinib (brand name: Vizimpro, company: PFIZER, New York City, the USA) and osimertinib (brand name: Tagrisso, company: ASTRAZENECA,London, the UK)), *ALK* (crizotinib (brand name: Xalkori, company: PF PRISM CV, Netherlands), alectinib (brand name: Alecensa, company: HOFFMANN-LA ROCHE, Basel, Switzerland), brigatinib (brand name: Alunbrig, company: TAKEDA PHARMS USA, Lexington, the USA), ceritinib (brand name: Zykadia, company: NOVARTIS, Basel, Switzerland), and lorlatinib (brand name: Lorbrena, location and company: PFIZER, New York City, the USA), *ROS1* (crizotinib (brand name: Xalkori, company: PF PRISM CV, Netherlands), lorlatinib (brand name: Lorbrena, company: PFIZER, New York City, the USA), entrectinib (brand name: Rozlytrek, company: GENENTECH INC, Pennsylvania, the US) and brigatinib (brand name: Alunbrig, company: TAKEDA PHARMS USA, the USA), *RET* (pralsetinib (brand name: Gavreto, company: GENENTECH INC, Pennsylvania, the US) and selpercatinib (brand name: Retevmo, company: LOXO ONCOLOGY INC, Massachusetts, the USA)) (123, 125–127). Some targets such as *HER2, KRAS, BRAF, NTRK*, and *MET* in the clinical trials benefit from the development of genomic profiling (128). The drugs target *HER2* mainly including TKIs (pyrotinib and tucatinib), mono-antibody (trastuzumab), and antibody–drug conjugates (trastuzumab deruxtecan) (129, 130), target *KRAS* contain adagrasib (MRTX849) and sotorasib (AMG510) (122), target BRAF (dabrafenib plus trametinib) (*NCT04452877 (first posted: 1 July 2020)*), target *NTRK* (larotrectinib and entrectinib) (*NCT02576431 (first posted: 15 October 2015), NCT02568267 (first posted: 5 October 2015)*), and target *MET* (crizotinib) (*NCT04084717 (first posted: 10 September 2019)*). The drugs approved by FDA significantly improved the OS of patients, such as gefitinib that improved the median PFS (mPFS) by almost 10.8 months, erlotinib increased mPFS by nearly 14 months, afatinib improved PFS by approximately 48 months, and dacomitinib increased mPFS up to 14.7 months (131–133). The mPFS of patients after taking osimertinib increased 18 months (134). The mPFS of patients with *ALK*-positive or *ROS-1*-positive NSCLC was increased 8.2 months after taking crizotinib and the OS was up to 114 months after taking lorlatinib (135, 136). The mPFS of *ALK*-positive metastatic NSCLC patients improved by 34.8 months after taking alectinib, and 7.8 months for ceritinib (135, 137). Brigatinib for NSCLC patients with *ALK-positive, ROS-1-positive, or EGFR* mutation-positive could also improve PFS by almost 11.0 months (138). Pralsetinib and selpercatinib for NSCLC patients with metastatic *RET* fusion- positive could also improve mPFS by almost 17.1 months and 16.5 months, respectively (139).

#### 4.2.2 Drugs still in preclinical and clinical trials

There are some drugs that are still in clinical trials, but also show significant effects on prolonging the OS of NSCLC patients. These drugs could give more hope to patients. For example, pyrotinib for advanced NSCLC with *HER2* mutation was proved to prolong the PFS of patients for 6.9 months and the median OS for 14.4 months in clinical trial (*NCT02834936 (first posted: 15 July 2016)*). Moreover, the new biomarkers found in the preclinical stage also provide targets for the treatment of lung cancer, for example, the mutations of the *PIK3CA* gene (140) and overexpression of VEGF in lung cancer driven by smoking (141).

Even though targeted therapy could produce high RR and improve the OS of patients, the special targets, such as *EGFR, ALK*, and *ROS1*, only account for a very small part (<20%) in the lung cancer patients (142). Hence, there is an urgency to develop more nonspecific therapies so they can be used to treat more lung cancer patients. The high cost of targeted therapy in the clinical treatment of lung cancer still limits its usage (143). Additionally, there are also some questions such as chemo-resistance in clinical therapy with the wide use of targeted drugs. The mechanism of acquired resistance in NSCLC after treatment with *EGFR* TKIs for several months mainly includes the hepatocyte growth-factor receptor amplification. Currently, deoxypodophyllotoxin (DPT) has been reported to reduce the resistance of HCC827GR cells by targeting *EGFR* and the hepatocyte growth-factor receptor, and induce apoptosis. This study could provide a combination therapy for the use of *EGFR* TKIs to reduce acquired resistance in the clinic (144). Furthermore, there are other therapies combined with targeted drugs that are in clinical trial.

The combination of erlotinib (an *EGFR* inhibitor) and bevacizumab (a monoclonal antibody targeting VEGF) could prolong the PFS of NSCLC patients (*NCT02759614 (first posted: 3 May 2016)*) (145). This reveals the probability of VEGF and *EGFR* double inhibition in the untreated metastatic *EGFR*-mutated NSCLC. Apatinib (a *VEGFR* inhibitor) plus gefitinib (a first-generation *EGFR* TKI) could prolong the mPFS for 19.2 months in advanced NSCLC with *EGFR* mutation, but this combination therapy also has some side effects and the quality of life (QoL) did not change (*NCT02824458 (first posted: 6 July 2016) (* 146). The use of osimertinib (a third generation of *EGFR* TKI) is usually accompanied by chemo-resistance in the terminal treatment of advanced *EGFR-*mutated NSCLC patients; the reason maybe because the second-site mutations appear in the *EGFR*. Therefore, osimertinib plus dacomitinib (a pan-HER inhibitor) could reduce drug resistance appearing in therapy, in a phase I/II trial *(NCT03810807 (first posted: 22 January 2019)*) *(* 147). Moreover, the combination of osimertinib and navitoclax (an inhibitor of BCL-2 that could increase apoptosis and reduce chemo-resistance) was feasible in patients with *EGFR*-mutated NSCLC in a phase IB trial (*NCT02520778 (first posted: 13 August 2015)*) *(* 148). The inhibitors targeting *KRAS* mostly through targeting *KRAS p. G12c*, for example, AMG510 and MRTX849 are still in the clinical study (149). AMG15 was used to treat patients with advanced metastatic NSCLC patients with *KRAS p. G12c* mutation in a phase 3 study (*NCT04303780, first posted: 11 March 2020*). MRTX849 showed better results in NSCLC patients, but had more side effects compared with AMG510. However, for clinical studies such as *NCT04613596 (first posted: 3 November 2020), NCT04685135 (first posted: 28 December 2020), and NCT04330664 (first posted: 1 April 2020)* results are yet to be obtained. AMG510 had already been approved by FDA. ARS-1620, an inhibitor of *KRAS* p. G12c is still in the preclinical stage but shows better anti-cancer ability in NSCLC through targeting his95 amino acid on *KRAS* p. G12c (150).

However, there are also some combination therapies that did not reach the expected results. For example, the combination of binimetinib (a *MEK* inhibitor), cisplatin, and pemetrexed did not improve anti-tumor activity compared with the chemotherapy of cisplatin and pemetrexed in advanced NSCLC with *KRAS* mutation (151). In a phase II study (*NCT03133546 (first posted: 28 April 2017)*), the combination of osimertinib (an *EGFR* TKI) and bevacizumab (a monoclonal antibody targeting VEGF) did not prolong the PFS in patients with advanced NSCLC with *EGFR and T790M* mutations; instead, the side effects increased (152). However, these trials also provide a guidance for clinical therapy (**Table 2**).

**Table 2:** Drugs used in chemotherapy and targeted therapy.

Therapy	Compounds	Application	Phase	NCT number	Improved survival time
Chemotherapy	RO4929097 plus erlotinib	Advanced NSCLC	Phase I, phase II	*NCT01193881NCT01193868*	PFS: 5 years
Chemotherapy	Sulforaphane plus Cisplatin and doxorubicin	NSCLC	Preclinical		
Chemotherapy	AKBA plus cisplatin	NSCLC	Preclinical		
Chemoradiotherapy plus immunotherapy	conventional chemoradiotherapy (platinum-based chemotherapy and radiotherapy) plus durvalumab	Stage III NSCLC	Phase III	*NCT02125461*	PFS: 3 years, OS: 4 years
Targeted therapy	Gefitinib, erlotinib, afatinib, and dacomitinib	NSCLC with *EGFR* mutation (exon 19 deletions, exon 21 substitution mutations)	Approved		mPFS: 10.8 months (gefitinib) (131), mPFS: 10-14 months (erlotinib) (132), PFS: 48 months (afatinib), and mPFS: 14.7 months (dacomitinib) (133)
Targeted therapy	Osimertinib	Metastatic NSCLC with *EGFR* mutation (*T790M* mutation)	Approved		mPFS: 18 months (134)
Targeted therapy	Crizotinib, lorlatinib	*ALK*-positive or *ROS-1*-positive NSCLC	Approved		mPFS: 8.2 months (crizotinib) (135), OS: 114.0 months (lorlatinib) (136)
Targeted therapy	Alectinib, ceritinib	*ALK*-positive metastatic NSCLC	Approved		mPFS: 34.8 months (alectinib) (137), mPFS: 7.8 months (ceritinib) (135)
Targeted therapy	Brigatinib	NSCLC with *ALK-positive, ROS-1-positive, or EGFR* mutation-positive	Approved		PFS: 11.0 months (138)
Targeted therapy	Dabrafenib plus trametinib	*BRAF V600E* Mutant metastatic NSCLC	Phase II	*NCT04452877*	Completion date: 28 December 2023
Targeted therapy	Larotrectinib	metastatic NSCLC harboring an *NTRK* fusion without acquired mutation for resistance	Phase II	*NCT02576431*	Completion date: 29 August 2025
Targeted therapy	Entrectinib	Metastatic *ROS-1*-positive NSCLC	Approved		
Targeted therapy	Entrectinib	NSCLC harboring an *NTRK1/2/3, ROS-1*, or *ALK* gene fusion	Phase II	*NCT02568267*	Completion date: 1 April 2025
Targeted therapy	Crizotinib	*ROS-1* or *MET* mutated NSCLC	Phase II	*NCT04084717*	Completion date: June 2025
Targeted therapy	Pralsetinib, selpercatinib	metastatic *RET* fusion-positive NSCLC	Approved		mPFS: 17.1 months (pralsetinib), mPFS,16.5 months (selpercatinib) (139)
Targeted therapy	Pyrotinib	Advanced NSCLC with *HER2* mutation	Phase II	*NCT02834936*	PFS: 6.9 months, median OS: 14.4 months
Targeted therapy	Tucatinib	*HER2*-expressing NSCLC	Phase II	*NCT05091528*	Completion date: April 2023
Targeted therapy	Trastuzumab	NSCLC	Phase II	*NCT00758134*	No results posted
Targeted therapy	Trastuzumab deruxtecan	*HER2*-mutated metastatic NSCLC	Phase II	*NCT04644237*	Completion date: September 2023
Targeted therapy	Adagrasib	NSCLC harboring the *KRASG12C* mutation	Phase III	*NCT04685135*	Completion date: July 2024
Targeted therapy	Sotorasib	Stage IV NSCLC with *KRAS p.G12C* mutation	Phase II	*NCT04933695*	Completion date: 21 February 2028
Targeted therapy	DPT plus gefitinib	NSCLC	Preclinical		
Targeted therapy plus antiangiogenic therapy	Erlotinib plus bevacizumab	Untreated metastatic *EGFR*-mutated NSCLC	Phase III	*NCT02759614*	No results posted
Targeted therapy plus antiangiogenic therapy	Gefitinib plus apatinib	Advanced NSCLC with *EGFR* mutation	Phase III	*NCT02824458*	mPFS: 19.2 months
Targeted therapy	Osimertinib plus dacomitinib	Advanced *EGFR* mutant lung cancer	Phase I/II	*NCT03810807*	Completion date: January 2023
Targeted therapy	Osimertinib and navitoclax	*EGFR*-mutated NSCLC	Phase IB	*NCT02520778*	Completion date: 30 July 2022

### 4.3 Antiangiogenic therapy

The abnormal growth of tumor is always accompanied by angiogenesis to supply nutrition for the cancer (153). Molecular markers such as hypoxia-inducible factor (HIF), vascular endothelial growth factor (VEGF), and VEGF receptor (VEGFR) play an important role in this process, and the most used targets are VEGF and VEGFR in cancer therapy (154). In addition, VEGF in the tumor microenvironment (TME) could inhibit the immune reaction of the immune cells. Therefore, VEGF inhibitors could also increase the capacity of immune cells (155). This reminds us that antiangiogenic therapy could combine with immunotherapy to benefit cancer patients. In clinical therapy, using antiangiogenic strategy usually involves two ways, namely, using the antibody to block the reaction between VEGF and VEGFR and using TKIs to inhibit the VEGFR and corresponding signaling pathways (156).

Bevacizumab (brand names: Avastin, Mvasi, Zirabev, company: GENENTECH, AMGEN INC, PFIZER INC), a monoclonal antibody targeting VEGF, has been approved by FDA and could play a role in the NSCLC treatment. The most widely explored use of bevacizumab is in combination therapy. Bevacizumab could increase the PFS (up for 4.4 months) and median OS compared with chemotherapy, but there is no difference of OS between the two therapies (*NCT00318136 (first posted: 26 April 2006), NCT00806923 (first posted: 11 December 2008)*), and the combination therapy of antiangiogenic therapy plus chemotherapy (bevacizumab plus cisplatin and gemcitabine) could prolong the median OS more than 13 months (157). Bevacizumab and atezolizumab are confirmed to be a potential therapy for the non-squamous NSCLC patients with higher *PD-L1* expression (≥50%) but without *EGFR/ALK/ROS1* mutations, in a phase II study (*NCT03836066 (first posted: 11 February 2019)*) (158). In a phase III trial (*NCT02366143 (first posted: 19 February 2015)*), bevacizumab combined with immunotherapy atezolizumab and chemotherapy (carboplatin and paclitaxel) could act as the first-line treatment in NSCLC patients with *KRAS* and *STK11* mutations and/or *STK11, KEAP1, TP53* mutations and/or high *PD-L1* expression (≥50%) (159), and the PFS of patients was up to 29 months and the OS of patients was prolonged by almost 53 months. Moreover, the biosimilars of bevacizumab, such as FKB238 and LY01008 have also shown the same efficiency and safety in non-squamous NSCLC patients, and the patients’ PFS and OS were almost 30 months after taking these drugs. These trials were in the phase III (*NCT02810457 (first posted: 23 June 2016), NCT03533127 (first posted: 22 May 2018)*) *(* 160, 161).

VEGFR includes VEGFR1, VEGFR2, and VEGFR3. Even though VEGFR1 and VEGFR2 correlated with angiogenesis, the affinity between VEGFR1 and VEGF is relatively weak. In addition, VEGFR3 regulates lymphangiogenesis (162, 163). Therefore, the target used in anti-angiogenesis in the clinic is usually VEGFR2. Apatinib, a VEGFR2 TKI, has been confirmed to significantly increase the PFS in advanced NSCLC patients with *EGFR* mutation combined with gefitinib, but the QoL did not change (*NCT02824458 (first posted: 6 July 2016)*) *(* 146). In a phase IB clinical trial (*NCT04670107 (first posted: 17 December 2020)*), anlotinib, a multitarget receptor of TKI, plus PD-1 inhibitor camrelizumab showed some efficiency in advanced NSCLC patients who are resistant to the first-line therapy (164).

### 4.4 Immunotherapy

Immunotherapy in NSCLC usually uses some antibodies to block the recognize between the antigens in immunocytes and ligands in tumor cells (165). Immune checkpoint inhibitors (ICIs) are usually used in advanced and metastatic NSCLC (166). The most widely used targets in NSCLC include cytotoxic T-lymphocyte-associated protein 4 (CTLA-4), programmed death receptor 1 (PD-1), and programmed death-ligand 1 (PD-L1) (167).

The corresponding monoclonal antibodies that are well-developed include anti-CTLA-4 antibody (ipilimumab (brand names: Yervoy, company: BRISTOL MYERS SQUIBB)), anti-PD-1 antibodies (pembrolizumab (brand names: Keytruda, company: MERCK SHARP DOHME), and nivolumab (brand names: Opdivo, company: BRISTOL MYERS SQUIBB)), and anti-PD-L1 antibodies (atezolizumab (brand names: Tecentriq, company: GENENTECH INC), durvalumab (brand names: Imfinzi, company: ASTRAZENECA UK LTD), and avelumab (brand names: Bavencio, company: EMD SERONO INC)) (168). Recently, immunotherapy in NSCLC has been further developed and plays an even more important role in NSCLC. The drugs approved by FDA in immunotherapy could improve the survival of patients. For example, ipilimumab could improve the patients’ PFS up to 0.84 years, and these are patients normally with PD-L1 overexpression and no *EFGR or ALK* mutation (169). Patients with metastatic NSCLC with high PD-L1 expression (≥50%) and without EGF) or *ALK* mutation could improve mPFS for 10.3 months and median OS for 15.5 months after taking pembrolizumab and atezolizumab (170, 171). Patients with metastatic NSCLC with *EGFR- or ALK-positive* mutation could acquire a better mPFS (4.2 months) and median OS (14.4 months) (172). Avelumab could improve the PFS almost 907 days in patients with PD-L1 positive and after failure of a platinum-based doublet (*NCT02395172 (first posted: 20 March 2015)*). Durvalumab was proved to increase the PFS up to 907 days and OS up to 1,420 days after chemotherapy and radiotherapy failed for patients with unresectable stage III NSCLC in a phase III trial (*NCT02395172 (first posted: 20 March 2015)*). Sugemalimab, an anti-PD-L1 monoclonal antibody, was used in stage IV NSCLC (*NCT03789604 (first posted: 28 December 2018)*) *(* 173). In a phase III trial, sugemalimab had the same OS and better PFS compared with durvalumab (174). Toripalimab, an anti-PD-1 antibody, was reported to play a role in the limited-stage small cell lung cancer, which has no reaction to the current chemotherapy (*NCT04418648 (first posted: 5 June 2020)*). In a phase II study (*NCT04304248 (first posted: 11 March 2020)*), toripalimab combined with platinum-based doublet chemotherapy could produce higher MPR/pCR rates in stage III NSCLC (175).

**Figure 3:**
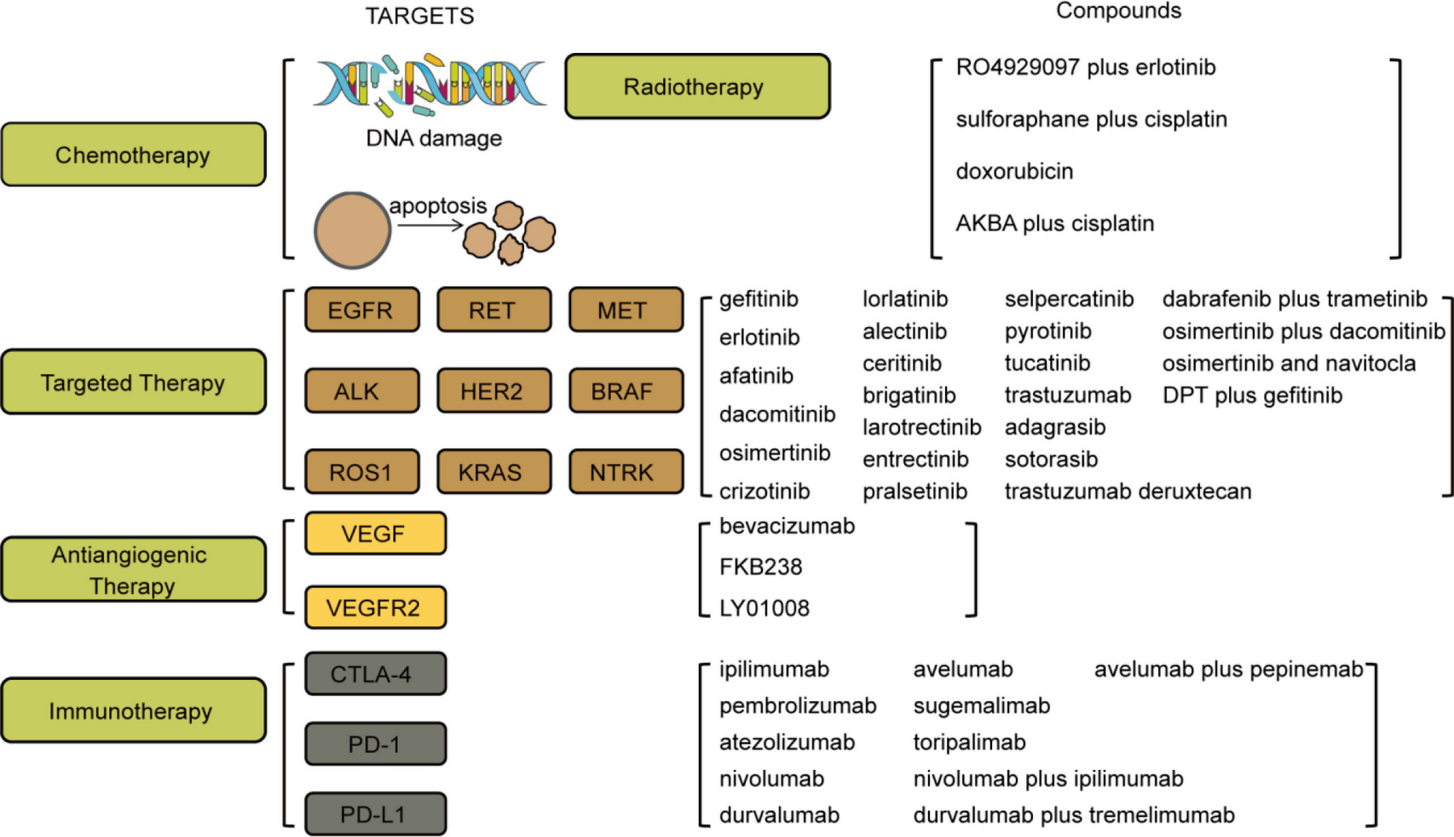
Targets and compounds in the treatment of NSCLC. Therapies in use are chemotherapy, targeted therapy, antiangiogenic therapy, and immunotherapy. Targets in chemotherapy include DNA damage and apoptosis. Targets in targeted therapy are EGFR, RET, MET, ALK, HER2, BRAF, ROS1, KRAS, and NTRK. Targets in antiangiogenic therapy are VEGF and VEGFR2. Targets in immunotherapy are CTLA-4, PD-1, and PD-L1. The corresponding drugs or compounds are listed on the right.

Other immunotherapies for NSCLC usually takes combination therapy and not limited to the monoclonal antibody alone. The combination therapy including immunotherapy plus chemotherapy (chemo-immunotherapy), immunotherapy plus radiotherapy, chemo-immunotherapy and radiotherapy. In a phase III trial (*NCT02492568 (first posted: 8 July 2015), NCT02444741 (first posted: 14 May 2015)*), pembrolizumab (an anti-PD-1 antibody) with radiotherapy could significantly increase the outcome of metastatic NSCLC patients (176). In a phase III trial (*NCT02477826 (first posted: 23 June 2015)*), nivolumab (an anti-PD-1 antibody) plus ipilimumab has a long-term efficacy in patients who have advanced NSCLC (177), but this combination could not prolong the OS in extensive-disease SCLC patients, in a phase III trial (*NCT02538666 (first posted: 2 September 2015)*) *(* 178). Furthermore, nivolumab plus ipilimumab combined with chemotherapy such as platinum doublet (179) or two cycles of chemotherapy (180) could extend the OS of patients in advanced stages compared with chemotherapy alone. Durvalumab, an anti-PD-L1 antibody, also combined with other monoclonal antibodies, chemotherapy or radiotherapy, has a better outcome compared with durvalumab alone. The most common combination is durvalumab and tremelimumab (an anti-CTLA-4 antibody) plus radiotherapy or chemotherapy. In a phase II study (*NCT03373760 (first posted: 14 December 2017)*), the combination of durvalumab and tremelimumab has some activity in patients with advanced NSCLC with resistance to PD-(L)1 therapy, and the OS of patients was 7 months (181). Durvalumab and tremelimumab plus chemotherapy such as platinum had no marked improvement on the OS of patients with advanced NSCLC (182). Furthermore, durvalumab and/or tremelimumab plus radiotherapy improves the efficacy and tolerance of NSCLC patients who are not suited for chemotherapy (*NCT05000710 (first posted: 11August 2021)*). Therefore, the optimum combination with durvalumab still needs more research to explore. However, current research also provides an option for the patients. Camrelizumab is an investigational PD-L1 inhibitor. The combination therapy involving camrelizumab has also been a research interest. In a phase III trial (*NCT03668496 (first posted: 12 September 2018)*), camrelizumab plus chemotherapy such as carboplatin and paclitaxel could dramatically extend the PFS (9.1 months) and median OS (18.2 months) in patients with advanced NSCLC (183). The same result was also found in another phase III trial (*NCT03134872 (first posted: 1 May 2017)*). The combination of camrelizumab and chemotherapy including carboplatin and pemetrexed could also ameliorate the mPFS (11 months) of NSCLC patients without *EGFR and ALK* mutations (184). More interestingly, in a phase Ib/II study (*NCT03268057 (first posted: 31 August 2017)*), pepinemab that mainly treats Alzheimer’s disease and Huntington’s disease in combination with avelumab (an anti-PD-L1 antibody) was proved well-tolerated in NSCLC patients (185). Even though the patients’ mPFS was only 8.4 weeks in this trial (**Table 3**), this clinical study provides a new option for the treatment of NSCLC (**Figure 3**).

**Table 3:** Drugs used in antiangiogenic therapy and immunotherapy.

Therapy	Compounds	Application	Phase	NCT number	Improved survival time
Antiangiogenic therapy	Bevacizumab	Unresectable, locally advanced or recurrent non-squamous NSCLC	Approved		PFS: 4.4 months (186)
Antiangiogenic therapy plus chemotherapy	Bevacizumab plus carboplatin and paclitaxel	Unresectable, locally advanced, recurrent or metastatic non-squamous NSCLC	Phase II	*NCT00318136*	No results posted
Antiangiogenic therapy plus chemotherapy	Bevacizumab plus cisplatin and gemcitabine	Locally advanced, metastatic, or recurrent non-squamous NSCLC	Phase III	*NCT00806923*	median OS>13 months
Antiangiogenic therapy plus Immunotherapy	Bevacizumab and atezolizumab	Non-squamous NSCLC patients with higher PD-L1 expression (≥50%) but without *EGFR/ALK/ROS1* mutations	Phase II	*NCT03836066*	Completion date: 30 January 2024
Antiangiogenic therapy plus immunotherapy and chemotherapy	Bevacizumab combined with atezolizumab and chemotherapy (carboplatin and paclitaxel)	NSCLC patients with *KRAS and STK11* mutations and/or *STK11, KEAP1, TP53* mutations and/or high PD-L1 expression	Phase III	*NCT02366143*	PFS: 29 months, OS: 53 months
Antiangiogenic therapy	FKB238, LY01008	Non-squamous NSCLC	Phase III	*NCT02810457, NCT03533127*	PFS: 30 months, OS: 30 months
Antiangiogenic therapy plus Immunotherapy	Anlotinib plus camrelizumab	Advanced NSCLC patients who are resistant to the first-line therapy	Phase IB	*NCT04670107*	No results posted
Immunotherapy	Ipilimumab	Metastatic NSCLC with PD-L1 overexpression and no *EFGR or ALK* mutation	Approved		PFS: 0.84 years (169)
Immunotherapy	Pembrolizumab, atezolizumab	Metastatic NSCLC with high PD-L1 expression (≥50%) and without EGF) or *ALK* mutation	Approved		mPFS: 10.3 months (170), median OS: 15.5 months (171)
Immunotherapy	Nivolumab	Metastatic NSCLC with *EGFR- or ALK-positive* mutation	Approved		mPFS: 4.2 months, median OS: 14.4 months (172)
Immunotherapy	Durvalumab	Unresectable stage III NSCLC after failed chemotherapy and radiotherapy	Phase III	*NCT02395172*	PFS: 907 days, OS: 1,420 days
Immunotherapy	Avelumab	PD-L1 positive, NSCLC after a failed platinum-based doublet	Phase III	*NCT02395172*	PFS: 907 days
Immunotherapy	Sugemalimab	Stage IV NSCLC	Phase III	*NCT03789604*	Completion date: 31 August 2024
Immunotherapy	Toripalimab	Limit-stage small cell lung cancer that has no reaction to the current chemotherapy	Phase III	*NCT04418648*	Completion date: 31 May 2024
Immunotherapy plus chemotherapy	Toripalimab plus platinum-based doublet chemotherapy	Stage III NSCLC	Phase II	*NCT04304248*	Completion date: 30 July 2026
Immunotherapy plus radiotherapy	Pembrolizumab plus radiotherapy	Metastatic NSCLC patients	Phase III	*NCT02492568, NCT02444741*	Completion date: 17 September 2022
Immunotherapy	Nivolumab plus ipilimumab	Stage IV NSCLC	Phase III	*NCT02477826*	Completion date: 30 August 2024
Immunotherapy	Durvalumab plus tremelimumab	Advanced NSCLC with resistance of PD-(L)1 therapy	Phase II	*NCT03373760*	OS: 7 months
Immunotherapy plus radiotherapy	Durvalumab and/or tremelimumab plus radiotherapy	Metastatic or locally advanced NSCLC	Phase II	*NCT05000710*	Completion date: December 2026
Immunotherapy plus chemotherapy	Camrelizumab plus chemotherapy such as carboplatin and paclitaxel	Stage IV squamous NSCLC	Phase III	*NCT03668496*	PFS: 9.1 months, median OS: 18.2 months
Immunotherapy plus chemotherapy	Camrelizumab and chemotherapy including carboplatin and pemetrexed	NSCLC patients without *EGFR and ALK* mutations	Phase III	*NCT03134872*	mPFS: 11 months
Immunotherapy	Avelumab plus pepinemab	Advanced NSCLC	Phase Ib/II	*NCT03268057*	mPFS: 8.4 weeks

## 5 Conclusion

Lung cancer is already becoming a worldwide threat to human life. NSCLC is a major type of lung cancer. In this review, we described the causes, biological features, (especially the mutations (*EGFR* mutation*, T790M* mutation, *RET rearrangements, MET* mutation, *ALK rearrangement, HER2* mutation, *BRAF* mutation, *ROS1 rearrangement, KRAS* mutation, *NTRK fusions*, and *PD-L1* mutation)), abnormal signaling pathways (MAPK/ERK, Bax/Bcl-2, FAK/AKT, ERK, PI3K/AKT/mTOR, JAK-STAT, RAS/MAPK, MDM2/p53, PTEN/PI3K/mTOR, MAPK/ERK, and NF-κβ signaling pathways), diagnostic methods (such as CT, PET scan, MRI scan, tissue biopsy, liquid biopsy, PCR, FISH, IHC, and NGS), and therapies for lung cancer, such as chemotherapy, chemoradiotherapy, targeted therapy, antiangiogenic therapy, immunotherapy, and some combination therapy. More specifically, we reviewed current drugs used in the clinic, including chemotherapy (RO4929097 plus erlotinib, sulforaphane plus cisplatin and doxorubicin, AKBA plus cisplatin), targeted therapy (gefitinib, erlotinib, afatinib, dacomitinib, osimertinib, crizotinib, lorlatinib, alectinib, ceritinib, brigatinib, dabrafenib plus trametinib, larotrectinib, entrectinib, pralsetinib, selpercatinib, pyrotinib, tucatinib, trastuzumab, trastuzumab deruxtecan, adagrasib, sotorasib, DPT plus gefitinib, osimertinib plus dacomitinib, osimertinib, and navitoclax), antiangiogenic therapy (bevacizumab, FKB238, LY01008), immunotherapy (ipilimumab, pembrolizumab, atezolizumab, nivolumab, durvalumab, avelumab, sugemalimab, toripalimab, nivolumab plus ipilimumab, durvalumab plus tremelimumab, avelumab plus pepinemab), combination therapy, such as chemoradiotherapy plus immunotherapy (conventional chemoradiotherapy (platinum-based chemotherapy add radiotherapy) plus durvalumab, toripalimab plus platinum-based doublet chemotherapy, camrelizumab plus chemotherapy such as carboplatin and paclitaxel, camrelizumab and chemotherapy including carboplatin and pemetrexed), targeted therapy plus antiangiogenic therapy (erlotinib plus bevacizumab, gefitinib plus apatinib), antiangiogenic therapy plus chemotherapy (bevacizumab plus carboplatin and paclitaxel, bevacizumab plus cisplatin and gemcitabine), antiangiogenic therapy plus immunotherapy (bevacizumab and atezolizumab, anlotinib plus camrelizumab), antiangiogenic therapy plus immunotherapy and chemotherapy (bevacizumab combined with atezolizumab and chemotherapy (carboplatin and paclitaxel)), and immunotherapy plus radiotherapy (pembrolizumab plus radiotherapy, durvalumab and/or tremelimumab plus radiotherapy). These diagnostic methods may also undergo further development accompanied by the application of deep learning artificial intelligence (AI) (187). From the drugs used in clinical treatment, we could find that combination therapy and targeted therapy or immunotherapy play an even more important role in the treatment of lung cancer. In addition, with increasing understanding of the pathogenesis of lung cancer and the development of sequencing, the novel targets in lung cancer could be found, and take a role in clinical drug development. Moreover, combination therapy with multi-types of treatment will benefit more patients with lung cancer.

## Author contributions

QG, LL, YF, YZ, and ZY reviewed the literature and drafted the article. ZC organized the figures and tables. QG, LL, ZY, and WZ finalized the paper and provided suggestions for improvement. All authors participated in designing the concept of this manuscript. All authors contributed to the article and approved the submitted version.

## Funding

The Medical Science and Technology Research Project of Henan Province (no. SBGJ202003010); the Medical Science and Technology Research Project of Henan Province (no. LHGJ20190675); the Doctoral Research Start-up Foundation of Henan Cancer Hospital.

## Conflict of interest

The authors declare that the research was conducted in the absence of any commercial or financial relationships that could be construed as a potential conflict of interest.

## Publisher’s note

All claims expressed in this article are solely those of the authors and do not necessarily represent those of their affiliated organizations, or those of the publisher, the editors and the reviewers. Any product that may be evaluated in this article, or claim that may be made by its manufacturer, is not guaranteed or endorsed by the publisher.
